# Fossil evidence for silica biomineralization in Permian lycophytes

**DOI:** 10.1093/nsr/nwae368

**Published:** 2024-10-21

**Authors:** Zhuo Feng, Qun Sui, Hai-Bo Wei, Jianbo Chen

**Affiliations:** Institute of Palaeontology, Yunnan Key Laboratory of Earth System Science, Yunnan Key Laboratory for Palaeobiology, MEC International Joint Laboratory for Palaeobiology and Palaeoenvironment, Yunnan University, China; Southwest United Graduate School, China; Institute of Palaeontology, Yunnan Key Laboratory of Earth System Science, Yunnan Key Laboratory for Palaeobiology, MEC International Joint Laboratory for Palaeobiology and Palaeoenvironment, Yunnan University, China; Institute of Palaeontology, Yunnan Key Laboratory of Earth System Science, Yunnan Key Laboratory for Palaeobiology, MEC International Joint Laboratory for Palaeobiology and Palaeoenvironment, Yunnan University, China; Institute of Palaeontology, Yunnan Key Laboratory of Earth System Science, Yunnan Key Laboratory for Palaeobiology, MEC International Joint Laboratory for Palaeobiology and Palaeoenvironment, Yunnan University, China

## Abstract

This paper reports the first in-situ fossil evidence for silica biomineralisation in Permian plants. The discovery reinforces the significant role that land plants have played in influencing the evolution of Earth systems in deep time.

The rise of land plants, in particular the early lycophytes, is considered to have significantly influenced the dramatic decline of atmospheric *p*CO_2_ in the Paleozoic via photosynthesis and subsequent organic carbon burial [1,2]. Silica biomineralization, a process during which plants uptake silicon (Si) from their environments and deposit it as extra- and intercellular components, can also reduce atmospheric *p*CO_2_ through accelerating the silicate weathering, which increases the Si flux into the ocean, and influences global carbon cycling [3,4]. A thorough investigation of living land plants shows that the extant lycophytes, particularly spikemoss (*Selaginella* Beauvoir), are some of the most effective Si accumulators in the plant kingdom, and thus, fossil spikemoss species have been hypothetically regarded as strong Si accumulators [5]. However, the silica biomineralization capability of early lycophytes is still somewhat a mystery due to the dearth of direct fossil evidence.

In this study, we document *in situ* phytoliths in nine forms of fossil spikemoss with exceptionally well-preserved cuticles from the upper Permian Xuanwei Formation in the Huangjiaochong coalmine (N 25°47′14′′, E 104°17′5′′) of Housuo Town, Fuyuan County, Qujing City, Yunnan Province, Southwest China (Notes S1 and S2; Fig. S1). The plant silica biomineralization of these late Permian (ca. 255-million-years old) lycophytes is demonstrated through a comparative study with extant spikemoss using transmitted light microscopy, scanning electron microscopy (SEM) and

energy-dispersive X-ray spectroscopy (EDX).

Based on their distinctive morphologies and anatomies, these Permian spikemoss leaves are separated into four morphotypes (Note S3; Figs S2 and S3). Each morphotype contains one to four leaf forms (Fig. 1a–j). Because none of these fossil leaves are attached to an axis, the different morphotypes or forms do not necessarily belong to an individual biological species, but may represent different parts (i.e. medium, lateral or axillary leaf) of the same species with varying morphology. These fossil leaves are assigned to spikemoss because all possess a single vein in the leaf, an acuminate apex and/or marginal hair-like structure, specialized papillate epidermal cells, and a relatively simple stomatal complex. Collectively, this combination of characteristics is unique for extant spikemoss and unknown from other land plants [6].

**Figure 1.**
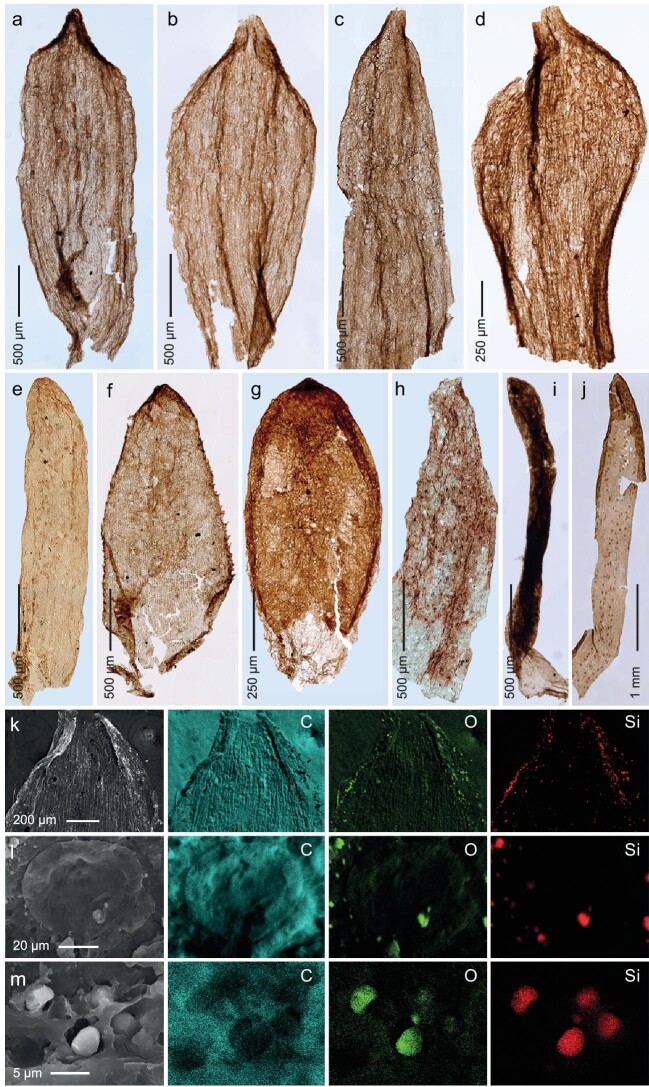
Late Permian spikemoss and SEM-EDX element maps of carbon (C), oxygen (O) and silicon (Si) from Southwest China. (a–d) Morphotype I. (e, f) Morphotype II. (i) Morphotype III. (j) Morphotype IV. (a, b) Leaves showing acuminate apices and sub-parallel margins. YNUPB10169 and YNUPB10170. (c) Leaf showing gradually tapered apex. YNUPB10171. (d) Leaf, broadest at the distal part. YNUPB10172. (e) Linear form showing blunt rounded apex, and sub-parallel margins. YNUPB10173. (f) Sub-triangular form showing hair-like structures on both margins. YNUPB10174. (g) Ovate form showing a short acute apex. YNUPB10175. (h) Gradually tapered form, covered by dense stomata. YNUPB10176. (i) Linear form, note the hair-like structures covering the whole leaf. YNUPB10177. (j) Lanceolate form, showing stomata zone above the midvein. YNUPB10178. (k) Leaf apex of morphotype I, note the positive correlation signal of O and Si on the outer surface of the leaf. (l) Stoma of morphotype I, showing phytoliths. (m) Silica bodies merged in the epidermal cells of morphotype II, showing no signal of C, but dense signals of O and Si.

High-resolution EDX analysis shows that carbon is the most common signal on all these fossil leaves, which is not surprising because the fossils are preserved as compressions. Dense positive correlation signals of Si and O (oxygen) are found on leaf surfaces (Fig. 1k) and stomata of a morphotype I leaf (Fig. 1l). Intensive positive correlation signals of Si and O are evident from the spheres and irregular plates that appear in the epidermal cells of a morphotype II leaf (Fig. 1m). These observations indicate that silica biomineralization is present in these Permian lycophyte leaves.

Extant spikemoss species (Notes S4–S7, Table S1) collected from the tropical region of Yunnan Province were comparatively investigated, given their environmental conditions are closely comparable to those of the Permian fossil site [7]. Morphologically, these extant species are characterized by prominent dimorphic leaves (Figs S4 and S5). Although the morphology and anatomy of extant species vary greatly, their spectral EDX patterns are similar to those of the fossil spikemoss. Variously shaped silica bodies ranging from spheroidal to plate-form are common on the leaf surface (Figs S6a and S7a) and stomata (Fig. S6b and d), and all species show positive correlation patterns of Si and O. However, intensive and positive correlation patterns attributed to Si and O are also exhibited by the papillate epidermal cells (Figs S6c and S7b) and stomata (Figs S6b and d, S7c and d), where there is no obvious phytolith. This phenomenon indicates that these cells are highly silicified.

The present Permian spikemoss leaves are remarkable because they are characterized by the presence of microscopic silica bodies (phytoliths). Phytoliths are commonly preserved in sediments after the decay of plants buried under diverse environmental conditions. Although the presence of dispersed phytoliths can be traced back to at least the Upper Devonian [8], knowledge of the parent plants of all previously documented pre-Cenozoic phytoliths is obscure. Thus, herein, we document the earliest record of *in situ* phytoliths in fossil plants. Our SEM-EDX investigations show that the distributional pattern of phytoliths in the present fossil spikemoss and extant spikemoss are somehow different from each other: the phytoliths in the extant spikemoss leaves are more regularly and evenly distributed than those in the fossil spikemoss leaves. This difference may represent an evolutionary trajectory of silica biomineralization in lycophytes during their long evolutionary history (Note S4).

It is unclear when and why non-vascular and vascular land plants obtained the capability of silica biomineralization. Our records indicate that Permian spikemoss exhibits strong Si-accumulation capability. Although the transportation and allocation of Si within extant plants have been extensively studied, the actual mechanism of silica biomineralization is still poorly understood [9]. Recently, Whalen *et al.* [10] proposed that the phytolith formation was an incidentally non-adaptive process among ancestral plants, but modified into adaptative phytolith deposits in later-diverging clades. The occurrence of phytoliths in Permian spikemoss, an early derived plant group, indicates that silica biomineralization is fundamentally similar to that in extant plants, supporting a comparable capability of silica biomineralization already present in late Paleozoic ancestors. Here, we speculate that this specific capability to accumulate Si resulted from a series of genomic novelties, which are considered important in the establishment of new features during the evolution of land plants [11]. Silica biomineralization significantly improves overall plant fitness, allowing vegetation to mitigate the negative effects of several external stresses, such as herbivory, pathogens and nutrient limitation.

The biomineralization of land plants plays a critical role in global Si cycling. Silicate weathering regulates atmospheric *p*CO_2_ by releasing dissolved silicon, which is partly taken up by plants and deposited as biogenic silica on land [12]. As rock weathering intensifies, plants exert more important biological control on Si cycling over millions of years [13]. Early terrestrial plants in the Paleozoic, such as bryophytes, lycophytes and some other vascular plants, are believed to have played a critical role in global Si cycling [14]. Our study provides the first direct fossil record of silica biomineralization of the early lycophytes, which resembles their living counterparts.

Bryophytes, a more primitive land-plant clade, also possess a high rate of silicon incorporation into their cellular bodies [5]. Phytolith investigation of extant bryophytes suggests that silica biomineralization in bryophytes could have a longer evolutionary history than lycophytes [15]. Although further testing is required, our study indicates that the acquisition of Si by early land plants may have played a significant role in shaping terrestrial ecosystems in the Paleozoic.

It is worth noting that unlike the fossil preparation methods used in the present study, all the previously described fossil spikemoss cuticles were macerated via the classical standard method using a high concentration of hydrofluoric acid, so that *in situ* silica bodies were dissolved during preparation (Note S1). The method we used to treat the fossil cuticles takes ten times as long as the traditional method, which may account for the present recovery of silica-bearing cuticles (Note S3). Nevertheless, using this modified technique, it may be possible to obtain phytoliths from other fossil plants. The application of this method on other fossil materials may provide a more comprehensive understanding of the physiological characteristics of fossil plants, and further elucidate how land plants influence the evolution of Earth surface systems through time.

## Supplementary Material

nwae368_Supplemental_File

## Data Availability

We are happy to share the fossil specimens upon request.
