# Supplementary material for: Fossil evidence for silica biomineralization in Permian lycophytes
Source: Natl Sci Rev. 2024 Oct 21;11(12):nwae368. doi: 10.1093/nsr/nwae368 (PMC11562828; doi:10.1093/nsr/nwae368)
Supplement: nwae368_Supplemental_File [file nwae368_supplemental_file.pdf]

Supplementary data for:

Fossil evidence for silica biomineralization in Permian lycophytes

Zhuo Feng[1,2]*, Qun Sui[1], Hai-Bo Wei[1] and Jianbo Chen[1]

[1] Institute of Palaeontology, Yunnan Key Laboratory of Earth System Science, Yunnan Key Laboratory for Palaeobiology, MEC International Joint Laboratory for Palaeobiology and Palaeoenvironment, Yunnan University, Kunming 650500, China

[2] Southwest United Graduate School, Kunming 650092, China

* Correspondence author. E-mail: zhuofeng@ynu.edu.cn

Note 1. Material and methods

The leaf anatomy of fossil spikemoss has been rarely documented, and cuticle-preserved specimens originate mainly from Carboniferous swamps. Four *Selaginella* leafy shoots, with epidermal structures, are presently known from the Pennsylvanian Period (late Carboniferous): *S. gutbieri* (Göppert) Thomas 2005, *S. stachygynandroiodes* (Geinitz) Thomas 2005, *S. zeilleri* (Halle) Thomas 2005 (Thomas, 2005), and *S. amasrae* Šimůnek and Thomas 2012 (Šimůnek and Thomas, 2012). These documentations provide a better understanding of the diversity and spatiotemporal distribution of the fossil taxon during the late Paleozoic, as well as their ecological significance (Bek et al., 2023). Silica bodies of these fossil spikemoss were not reported probably because of silicate-dissolving chemistries used to separate plant fossils from the rock matrix.

Hundreds of compressed, (sub)millimeter-sized spikemoss leaves were obtained by bulk maceration using hydrochloric acid (HCl) from the siltstone samples of the lower member of the Xuanwei Formation of Yunnan Province, Southwest China (Fig. S1a, b). Grayish silty mudstone and siltstone samples were split into small pieces of approximately 50 × 50 × 50 mm in dimension, placed into a beaker immersed with 30% HCl, and then heated at 65 °C in a fume hood for a minimum of 60 days when

the rock completely dissolved. The leaf remains were picked using a needle and treated using Schulze's reagent (~30% HNO_3 with a few crystals of $KClO_3$) for 5–10 days. Bleaching with 5% KOH for 5–10 minutes was followed by a distilled water wash to neutrality. The fossil cuticles were either dehydrated in pure glycerine and subsequently mounted in permanent glycerine-jelly slides for light microscopic examination, or dried naturally and mounted on metal stubs using carbon tapes for scanning electron microscopic (SEM) and energy-dispersive X-ray spectroscopy (EDX) investigations.

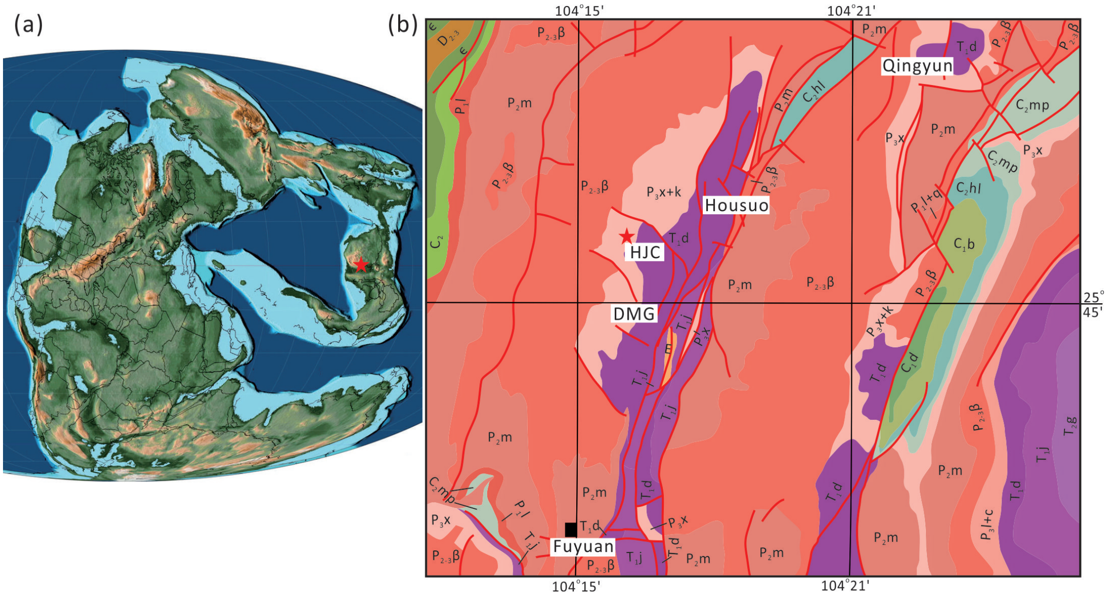

Figure S1. Paleogeographic (a, after Scotese, 2021) and geological (b) maps of the fossil locality (asterisk) in Fuyuan County of Yunnan Province, Southwest China that yielded the late Permian spikemoss leaves. DMG — Damogou Coalmine; HJC — Huangjiaochong Coalmine; Z — pre-Cambrian; ϵ — Cambrian; $D_{2\text{-}3}$ — Middle–Upper Devonian; C_1b — lower Carboniferous Baizuo Formation; C_1d — lower Carboniferous Datang Formation; C_2hl — upper Carboniferous Huanglong Formation; C_2mp — upper Carboniferous Maping Formation; P_1l — lower Permian Liangshan Formation; P_1q — lower Permian Qixia Formation; P_2m — middle Permian Maokou Formation; $P_{2\text{-}3}\beta$ — middle–upper Permian Emeishan Basalt; P_3x+k — upper Permian Xuanwei + Kayitou formations; P_3l+c — upper Permian Longtan + Changhsing formations; T_1d — Lower Triassic Dongchuan Formation; T_1j — Lower Triassic Jialingjiang Formation; T_2g — Middle Triassic Guanling Formation; Q — Quaternary.

The extant spikemoss specimens were collected from the tropical region of Yunnan Province, Southwest China. Specimens housed in the herbarium of Yunnan University were examined for taxonomic purposes. Extant spikemoss leaves were bleached by 5% NaOH for a minimum of 5 days, and then gently rinsed with distilled water. The epidermis and cuticles were either dehydrated in pure glycerine and subsequently mounted in permanent glycerine-jelly slides for light microscopic examination, or dried naturally and mounted on metal stubs using carbon tapes for SEM and EDX investigation.

Optical observations and photomicrographs were taken using a ZEISS Imager.Z2 transmitted light microscope equipped with a ZEISS Axiocam 512 color digital imaging system. SEM and EDX investigations were performed using a thermoscientific Quattro S SEM with a field emission gun. The fossil cuticle slides are housed in the Palaeobotanical Collections of the Institute of Palaeontology, Yunnan University, Kunming, China, under catalogue numbers YNUPB10169–183.

Note 2. Geology of fossil locality

The upper Paleozoic strata are well developed in eastern Yunnan Province, Southwest China. Tectonically this area is part of a stable intracratonic basin within the western Yangtze Block, located between the low-latitudinal Khangdian Oldland and the Cathaysian landmass in the eastern Tethys Ocean during the Paleozoic (Liu, 1990). The fossiliferous intercalated marine and non-marine deposits are a comprehensive basis for precise international stratigraphic correlation (Shen et al., 2019).

Fossil spikemoss leaf specimens were collected from the lower member of the Xuanwei (Hsuanwei) Formation in the Huangjiaochong coalmine of Housuo Town, Fuyuan County, Qujing City, Yunnan Province, Southwest China (Fig. S1a, b). The widespread upper Permian Xuanwei Formation is up to 274 m thick and consists of non-marine siliciclastic rocks. In the basal part the formation is dominated by

conglomerates unconformably overlying the mid–upper Permian Emeishan Basalt Formation. The rest of the formation is mainly composed of interbedded sand-, silt- and mudstone and up to 25 coal seams (Wang et al., 2019). The Xuanwei Formation is divided into a lower and an upper member, which are respectively Wuchiapingian and Changhsingian in age (Zhao et al., 1980). Fifty-five species of fossil plants belonging to twenty-nine genera have been identified from the Xuanwei Formation in Fuyuan County, constituting a typical Cathaysian *Gigantopteris* flora (Feng et al., 2017, 2023).

Paleomagnetic studies have indicated that the studied region was located at a paleolatitude of approximately 2.5° S during the late Permian to Early Triassic (Wang and Li, 1998). Therefore, the flora from the Xuanwei Formation of Southwest China is interpreted as representing an equatorial rainforest-like ecosystem (Feng et al., 2020).

Note 3. Full description of the late Permian spikemoss

Morphotype I contains the largest spikemoss leaves that are up to 5 mm long, lanceolate or spatulate in shape, and terminate in an acuminate tapering apex (Fig. 1a–d). Variation in this group consists of three forms with different shape. Form A shows a short apex and sub-parallel margins (Fig. 1a, b). Form B shows an elongate apex, and the lamina narrows distally (Fig. 1c). Form C possesses an elongate apex, with the broadest part of the leaf close to the apex (Fig. 1d). All three forms exhibit a broad, longitudinal coastal zone that divides the leaves into three almost equal zones. The leaf apices are rolled inward, forming tube-like structures. Leaf margins are mostly entire, with very few minute hair-like structures occasionally present on the apex (Fig. S2a). Stomata are found irregularly on both the upper (adaxial) and lower (abaxial) cuticles. Each stoma consists of two kidney-shaped guard cells, without subsidiary or neighbouring epidermal cells (Fig. S2b). The guard cells are generally highly cutinized, forming disc-like structures with prominently thickened rims, and are densely covered with spheroidal bodies of 2–10 μm diametre (Fig. S2c). Where

the spheroidal bodies have detached, distinct circular pits are present on the stomata (Fig. S2d, e). Epidermal cells are rectangular or sub-rectangular in shape, showing undulate anticlinal walls on both the upper and lower cuticles (Fig. S2f). Silica bodies (phytolith) are commonly present on the cuticle surface or embedded in the cuticle.

Morphotype II is the most abundant form with leaves up to 2 mm long, linear to ovate in shape, with blunt rounded or short pointed apices. Epidermal cells are elongate rectangular to isodiametric in shape. Morphotype II comprises four forms. Leaves in form A are linear, with stomata confined on the midvein; hair-like structures are generally present on the basal and mid leaf margins, rarely occur on the distal leaf margins (Figs. 1e, S2g). Form B is sub-triangular, with conspicuous hair-like structures at the leaf margins distributed from the base to the tip (Figs. 1f, S2h). In contrast, form C is ovate with a short acute pointed apex, and hair-like structures only present at the basal leaf margins (Fig. 1g). Form D is linear with an elongate apex, stomata densely concentrated on the lower leaf surface (Figs. 1h, S2i). The stomata of morphotype II leaves comprise two kidney-shaped guard cells, but without spheroidal bodies like those in the leaves of morphotype I (Fig. S3a). Specialized papillate epidermal cells commonly occur in the leaves of morphotype II (Fig. S3b, c). Spheroidal and irregular shaped silica bodies are common in the cuticles of morphotype II (Fig. S3d, e); pits are formed where the silica bodies have detached (Fig. S3f).

Morphotypes III and IV are rare in the cuticle assemblage. Morphotype III is linear with a blunt rounded apex, and bearing dense hair-like structures at the basal part of the leaf (Figs. 1i, S3g). Epidermal cells are only observed on the leaf base, showing rectangular outline. Morphotype IV is lanceolate with stomatal zones above the midvein on both the upper and lower cuticles (Fig. 1j). Epidermal cells of morphotype IV range from elongate rectangular to isodiametric in shape. Each stoma is characterized by two kidney-shaped guard cells surrounded by four to five epidermal cells (Fig. S3h, i). Irregular shaped silica bodies are common on the both

leaf morphotypes.

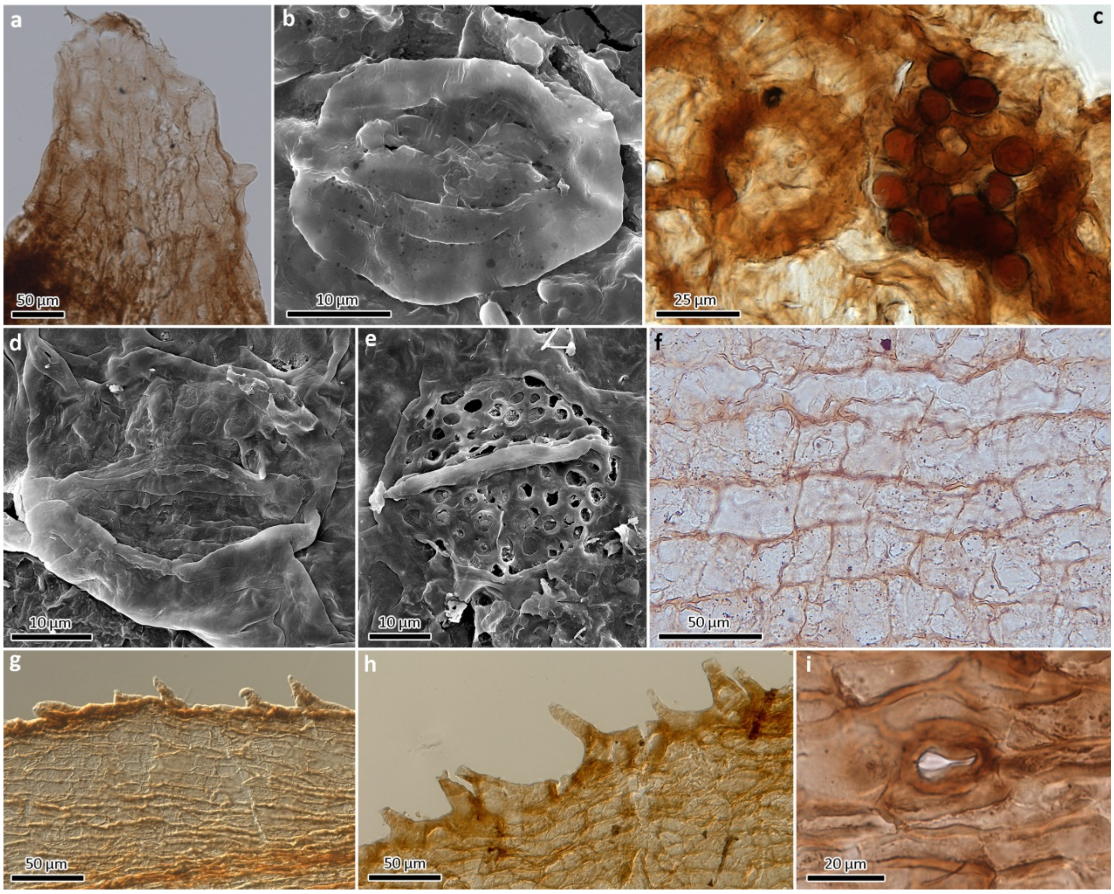

Figure S2. Epidermal anatomy of late Permian spikemoss (Morphotype I and II) from Southwest China. (**a**–**f**) Morphotype I. (**g**–**i**) Morphotype II. (**a**) The acuminate apex, showing short hair-like structure. YNUPB10179. (**b**) SEM image of a stoma; note the highly cutinized nature. (**c**) Showing two stomata, note the prominent rim and the spheres on the guard cells. YNUPB10180. (**d**) SEM image of a stoma, showing the guard cells with circular pits. (**e**) SEM image of a stoma, showing circular pits on the guard cells. (**f**) Showing rectangular-shaped epidermal cells; note the straight anticlinal walls and circular pits on the epidermal cells. YNUPB10181. (**g**) Showing hair-like structures on the leaf margin. YNUPB10182. (**h**) Showing hair-like structures on the leaf margin. YNUPB10174. (**i**) Showing a stoma surrounded by four epidermal cells. YNUPB10178.

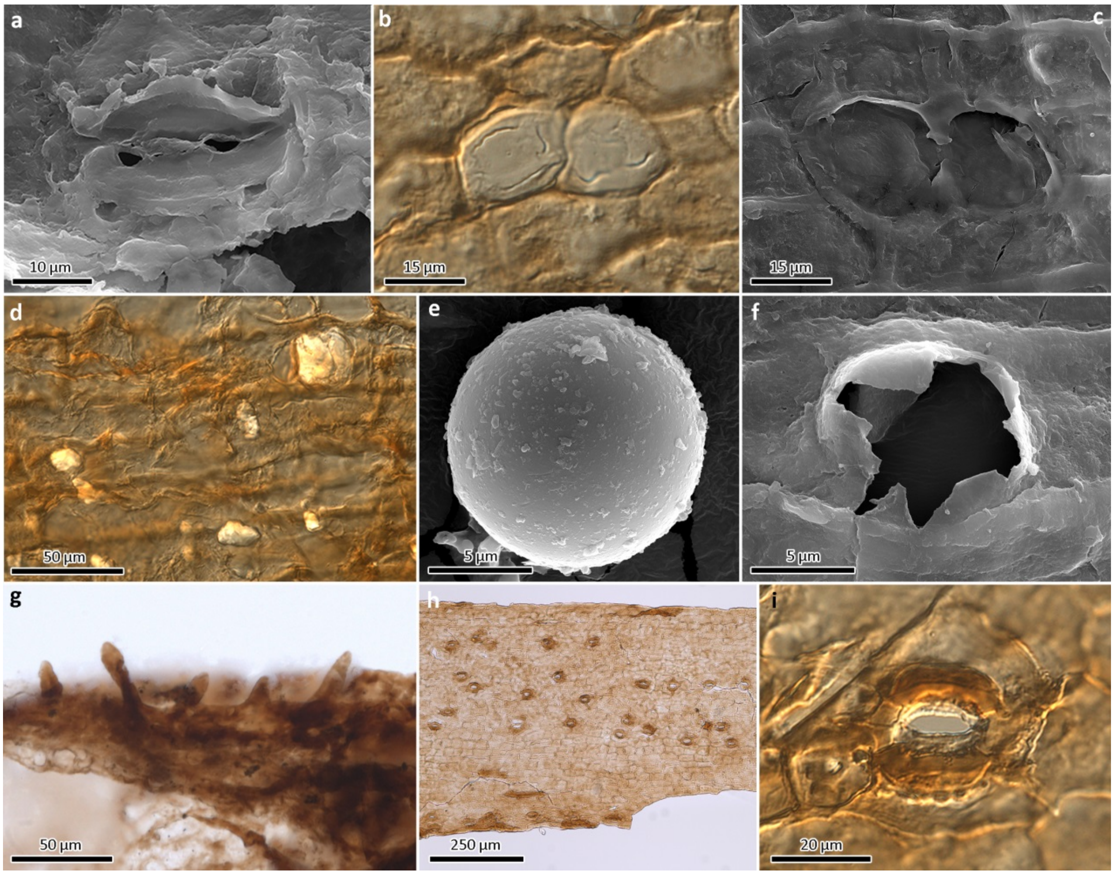

Figure S3. Epidermal anatomy of late Permian spikemoss (Morphotype II–IV) from Southwest China. (**a–f**) Morphotype II. (**g**) Morphotype III. (**h, i**) Morphotype IV. (**a**) SEM image showing details of the stomata; note the smooth surface. (**b**) Showing specialized papillate cells among the normal epidermal cells. YNUPB10183. (**c**) SEM image showing details of the specialized papillate cells. (**d**) Silica bodies embedded in the epidermal cells. (**e**) SEM image showing a spheroidal silica body derived from the leaf. (**f**) SEM image showing a spheroidal pit. (**g**) Hair-like structures on the basal part of the leaf. YNUPB10177. (**h**) Showing dense stomata above the midvein. YNUPB10178. (**i**) Showing the two kidney-like guard cells in a stoma. YNUPB10178.

Note 4. Extant spikemoss species analyzed in this study

The cosmopolitan *Selaginella* (spikemoss), the only genus of lycophyte family Selaginellaceae, comprises 600–800 extant species, representing one of the most species-rich genera among vascular plants (Weststrand and Korall, 2016; Zhou et al., 2016; Valdespino et al., 2018a). They are heterosporous, herbaceous, creeping to

prostrate, terrestrial or epipetric, and inhabit temperate, tropical, arctic and desert environments worldwide (Banks, 2009; Banks et al., 2011). Molecular dating and fossil record of the taxon indicate that the group's evolutionary history can be traced back to the Late Devonian–early Carboniferous (Matsunaga et al., 2017). This group of plants has maintained a striking morphological and reproductive stasis during its evolution over approximately 350–370 million years (Banks et al., 2011).

One of the most characteristic traits of *Selaginella* is the presence of specialized papillate epidermal cells. In a most recent thorough review, Lopes and Feio (2020) summarized various terms applied to this unique epidermal structure, i.e. warty sclereids (Dengler, 1980), warty fibers (Dahlen, 1988; Yang and Zhang, 2003), epidermal warts (Bienfait and Waterkeyn, 1974; Yang and Zhang, 2003), idioblasts (Cremers and Boudrie, 2007; Góes-Neto and Salino, 2018; Góes-Neto et al., 2015; Valdespino, 2017a), papillate cells (Góes-Neto et al., 2017), papillate idioblast-like cells (Valdespino et al., 2014), elongate papillate cells (Valdespino, 2015a, b, c; Valdespino et al., 2015), papillae (Valdespino, 2017a, b) and papillate idioblasts (Valdespino et al., 2018a, b).

Based on SEM and EDX spectroscopic analyses of 55 extant *Selaginella* species, Lopes and Feio (2020) confirmed that the specific epidermal structure showed a strong signal of Si, and subsequently the authors referred to these structures as silica bodies. However, though these structures show a strong signal of Si, they may not necessarily be silica bodies according to our experiments. It is more likely that these structures represent Si-rich papillae or silicified papillae. Due to the fact that, 1) the epidermal cells without papilla can also show a strong signal of Si; 2) the tip of the marginal teeth in some species can also be separated from the leaf, and show a strong signal of Si; and 3) the papillae of our fossil specimens did not show any signal of Si, we use papillae to describe the specific epidermal structures in the current study.

A total of 72 extant *Selaginella* species have been documented in China (Zhang

et al., 2013), of which 53 species (with a few variation forms) were recorded in Yunnan Province (Table S1) (Chu, 2006). The distribution, gross morphology, ecological traits and reproductive biology of the Chinese *Selaginella* species have been extensively investigated. By contrast, hitherto the epidermal anatomies of these species have been rarely researched (Yang and Zhang, 2003; Sun et al., 2006; Guo et al., 2012), though the history of their anatomical study can be traced back to the early 20th century (Hsü, 1937).

Among the *Selaginella* species in Yunnan Province, there are 21 species/forms only occurred in cold and temperate regions, 21 species/forms exclusively distributed in subtropical and tropical regions, whereas 15 species/forms have a wide range of habitats from temperate to tropical regions. All the tropical species show dimorphic leaves bearing entire margins (e.g. *S. helferi* Warb., Fig. S4), or prominent ciliated margins (e.g. *S. biformis* A. Braun ex Kuhn, Fig. S5).

Because environmental conditions may affect phytolith formation and phytolith properties in plants (Liu et al., 2016; Lopes and Feio, 2020), all extant spikemoss specimens illustrated herein were collected from the tropical regions (Menglun Town, Mengla County, Xishuangbanna Prefecture, Yunnan Province), where the environmental conditions are considered to be closely comparable to those of the Permian fossil site (Feng et al., 2020). Herbarium materials housed in Yunnan University have also been examined in this study for taxonomical purpose.

Note 5. Description of *S. helferi* Warb.

Selaginella helferi Warb. is mainly distributed in the subtropics and tropics, including Southwest China, India, Laos, Myammar, Thailand and Vietnam. The macromorphological and epidermal anatomical characteristics of this species (Chu, 2006; Sun et al., 2006; Zhang et al., 2013) are summarized here.

Selaginella helferi is terrestrial, evergreen, scandent, approximately 0.5–2 m high. The axillary leaves on the main stems are larger than those on branches,

orbicular or reniform, 3 × 2.8 mm in dimensions, biauriculate at base; axillary leaves on branches are symmetrical or slightly asymmetrical along both sides of axis, ovate-lanceolate or oblong, 1.4–2.5 × 0.8–1.2 mm, base biauriculate, margin entire. Leaves on both main stem and branches are arranged in four rows (Fig. S4a), including two rows of larger lateral leaves and two rows of smaller median leaves. The lateral leaves are asymmetrical; those on main stem are larger than those on branches; they are contiguous, spreading, oblong-falcate, 2.3–4.2 × 0.9–1.8 mm, apex acute or apiculate; basiscopic margin entire; acroscopic base with rounded auricle, not overlapping the axis, margin entire (Fig. S4b). The median leaves are asymmetrical, and those on the main stem are slightly larger than those on branches; they are commonly overlapping the axis at leaf apex, falcate, 1.2–2.5 × 0.3–1 mm, none-carinate, base oblique, margin entire, apex cuspidate (Fig. S4c).

In the lateral leaves, the epidermal cells are elongate rectangular, with sinuous anticlinal walls on the upper (abaxial) surface (Fig. S4d); but are isometric polygonal or subcircular, and have straight anticlinal walls on the lower (adaxial) surface of lateral leaves (Fig. S4e). Each stomatal complex consists of two kidney-shaped guard cells, which are commonly highly cutinized (Fig. S4f). There is one prominent papilla forming a dome-like structure on the centre of each epidermal cell on the lower leaf surface (Fig. S4g). Stomata are mainly distributed on the adaxial surface and on the midveins or closely along both sides of midveins (Fig. S4l).

In the median leaves the epidermal cells range from elongate rectangular to irregularly elongate in shape, and show sinuous anticlinal walls on the lower surface (Fig. S4h); they are isometric polygonal or subcircular and show straight anticlinal walls on the upper surface of lateral leaves (Fig. S4i). Each stomatal complex consists of two kidney-shaped guard cells, which are commonly highly cutinized (Fig. S4j). There is one prominent papilla forming a dome-like structure on the centre of each epidermal cell on the upper leaf surface (Fig. S4k). Stomata are mainly distributed on the upper surface and on the midveins or closely along both sides of the midveins

(Fig. S4m).

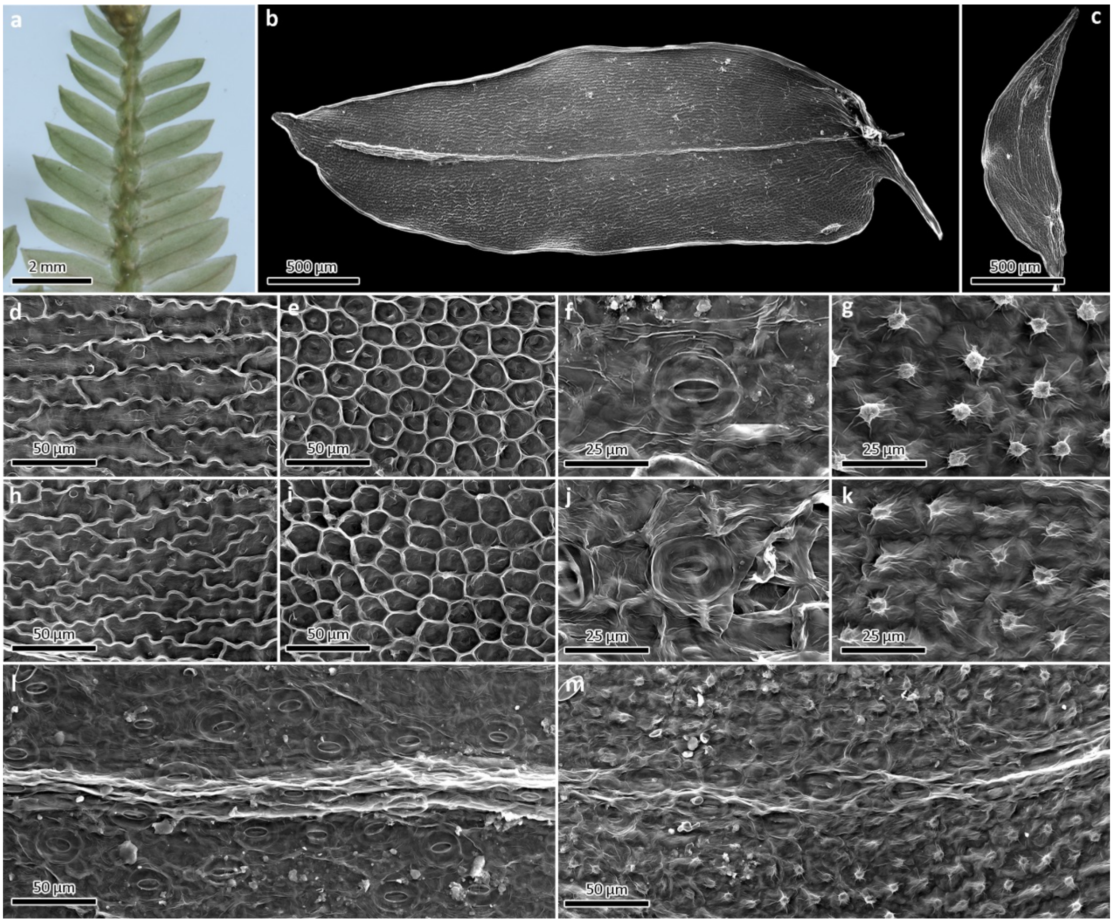

Figure S4. *Selaginella helferi* Warb. from the tropical region of Yunnan Province, Southwest China. (**a**) Light microscopic photograph, showing the distal part of a leafy shoot. (**b**) SEM image showing a lateral leaf. (**c**) SEM image showing a median leaf. (**d**) lateral leaf, SEM image showing the inner surface of the epidermal cells on the upper leaf surface. (**e**) Lateral leaf, SEM image showing the inner surface of the epidermal cells on the lower leaf surface. (**f**) Lateral leaf, SEM image showing a stoma on the lower leaf surface. (**g**) Lateral leaf, SEM image showing the papillate epidermal cells on the lower leaf surface. (**h**) Median leaf, image showing the inner surface of the epidermal cells on the lower leaf surface. (**i**) Median leaf, SEM image showing the inner surface of the epidermal cells on the upper leaf surface. (**j**) Median leaf, SEM image showing a stoma on the upper leaf surface. (**k**) Median leaf, SEM image showing the papillate epidermal cells on the upper leaf surface. (**l**) Lateral leaf, SEM image showing the lower leaf surface; note the stomata present on the midvein and

close to the midvein. (**m**) Median leaf, SEM image showing the upper leaf surface; note the stomata present on the midvein and close to the midvein.

Note 6. Description of *S. biformis* A. Braun ex Kuhn

Selaginella biformis A. Braun ex Kuhn is a species inhabiting shaded places or on rocks in (sub)tropical forests, including southern China, southern Japan, Vietnam, Laos, Myanmar, Thailand, India, Philippines, Indonesia, Malaysia and Sri Lanka. The macromorphological and epidermal anatomical characteristics of this species (Yang and Zhang, 2003; Chu, 2006; Zhang et al., 2013) are summarized here.

Selaginella biformis is terrestrial or epilithic, evergreen, erect or creeping, 0.15–0.45(–0.55) m high. The axillary leaves on branches are slightly asymmetrical, ovate-lanceolate or elliptic, 1.8–2.4 × 0.8–1.2 mm, base exauriculate, margin ciliolate at base. Leaves on both main stems and distal branches are arranged in four rows (Fig. S5a), including two rows of larger lateral leaves and two rows of smaller median leaves. The lateral leaves on branches are contiguous, ovate, 0.8–1.4 × 0.6–0.8 mm, not carinate, base obliquely cordate, margin very ciliolate, apex aristate (Fig. S5b, c). The median leaves on branches are contiguous or imbricate, slightly ascending, oblong-falcate or falcate, 1.8–3.2 × 1.2–1.6 mm, apex acute; basiscopic base rounded, margin subentire except base with a few hair-like structures; acroscopic base not enlarged, not overlapping the axis (Fig. S5d).

In the lateral leaves, hair-like structures are exclusively present on the acroscopic margin. The length of the hair-like structures is gradually shortened from the base to dentate at the tip (Fig. S5e, f). The epidermal cells are rectangular with sinuous anticlinal walls on the upper surface of the lateral leaves and the lower surface of the median leaves (Fig. S5g); but are isometric polygonal or subcircular with straight anticlinal walls on the lower surface of lateral leaves and on the upper surface of the median leaves (Fig. S5h). Prominent papillae and silicified cells are intensively present on the epidermal cells of both lateral and median leaves (Fig. S5i).

Stomata form a wide stomatal band along the midvein and are present on the lower surface of the lateral leaves (Fig. S5j) and the upper surface of the median leaves (Fig. S5k). Each stomatal complex consists of two kidney-shaped guard cells which are commonly highly cutinized (Fig. S5l); the dimension of the stomata on the lateral leaves appears larger than those on the median leaves.

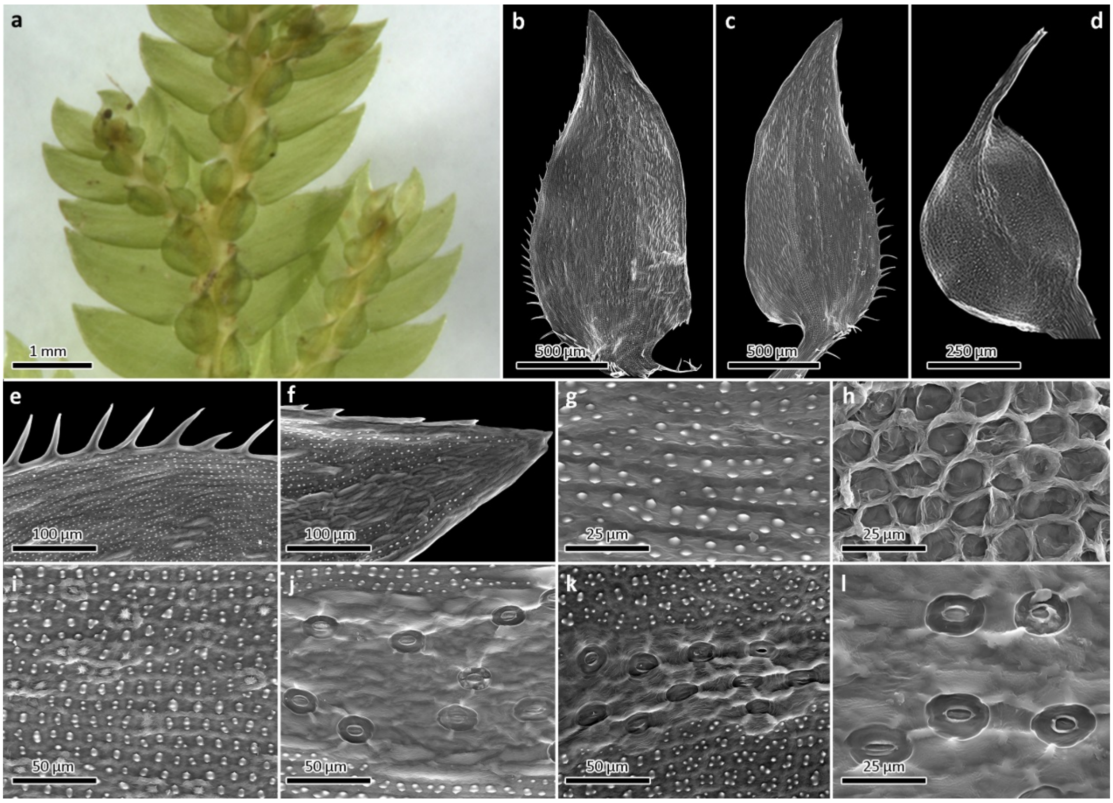

Figure S5. *Selaginella biformis* A. Braun ex Kuhn from the tropical region of Yunnan Province, Southwest China. (**a**) Light microscopic photograph, showing the branched distal part of a leafy shoot. (**b, c**) SEM images showing lateral leaves. (**d**) SEM image showing a median leaf. (**e**) Lateral leaf, SEM image showing the long hair-like structures on acroscopic margin of the lower part of a leaf. (**f**) Lateral leaf, SEM image showing the tapering leaf apex. (**g**) Lateral leaf, SEM image showing rectangular epidermal cells with papillae. (**h**) Median leaf, SEM image showing the inner surface of the epidermal cells on the upper leaf surface. (**i**) Lateral leaf, SEM image showing the papillae on the epidermal cells. (**j**) Lateral leaf, SEM image showing the stomatal band on the lower leaf surface. (**k**) Median leaf, SEM image showing the stomatal band on the upper leaf surface. (**l**) Lateral leaf, SEM image showing the stoma on the

lower leaf surface.

Note 7. High-resolution EDX analysis of extant spikemoss species from the tropics in Yunnan Province, Southwest China

Four extant spikemoss species were analyzed using EDX microscopy: *S. helferi*, *S. biformis*, *S. ciliaris* and *S. repanda*. Although the morphology and anatomy of these species varies greatly, their EDX characteristics are quite similar. Our analysis showed that various shaped phytoliths (silica bodies) are common on the leaf surface (Fig. 6a), which showed dense positive correlation signals of Si and O. Intense positive correlation signals of Si and O are also evident in the papillate cells (Figs. 6c, S7a, b) and stomata (Figs. 6b, S7c, d), where no phytolith was observed. This phenomenon indicates that these cells are highly silicified.

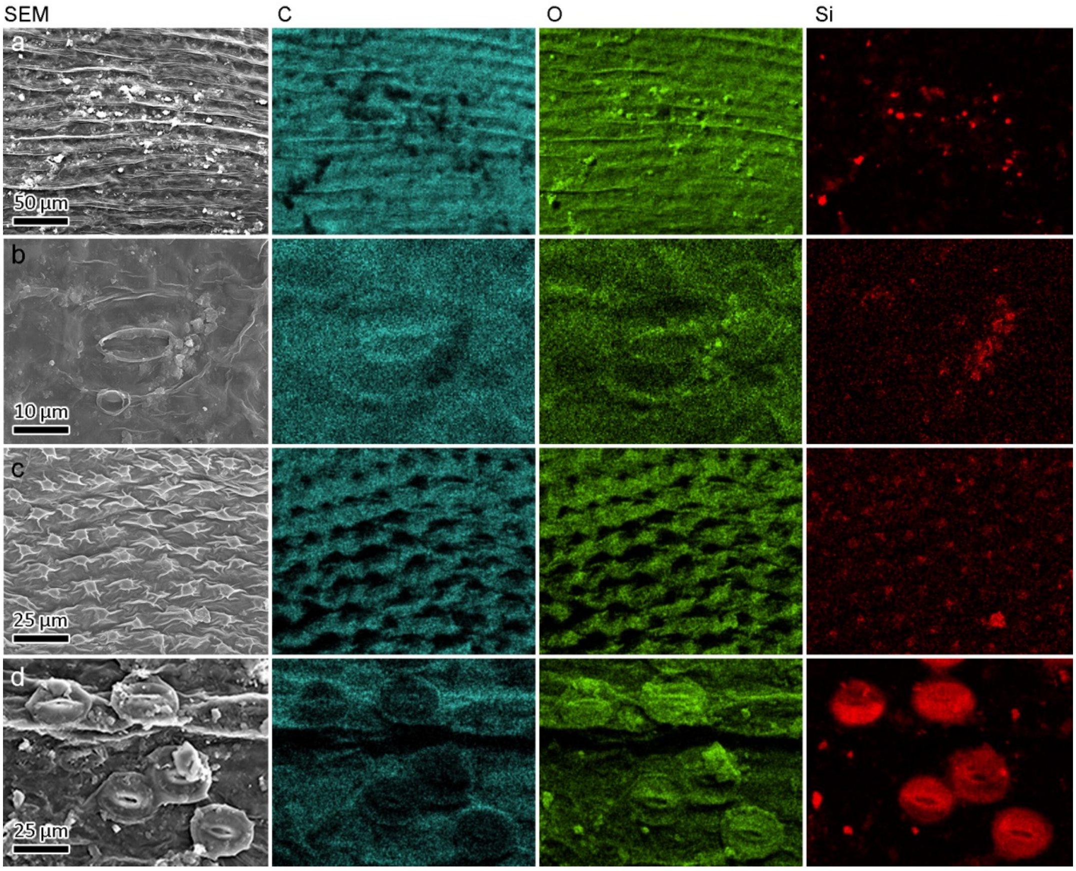

Figure S6. SEM-EDX analysis of *Selaginella helferi* Warb. from Southwest China, with successive element maps of carbon (blue), oxygen (green) and silicon (red). (a) Various shaped phytoliths on the leaf surface. (**b**) Phytoliths on the stomatal surface.

(**c**) Idioblast cells showing dense signals of O and Si. (**d**) Stomata showing dense signals of O and Si.

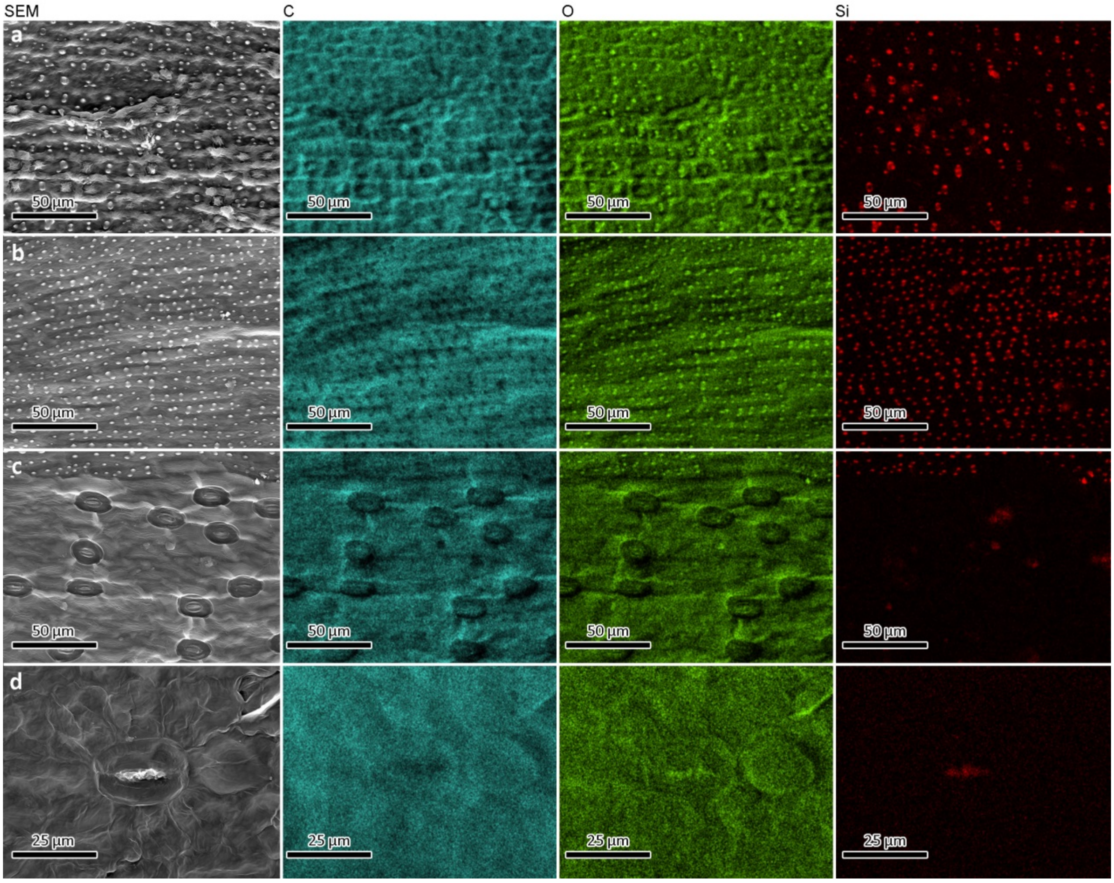

Figure S7. SEM-EDX analysis of extant spikemoss from the tropical region of Yunnan Province, Southwest China, with successive element maps of carbon (blue), oxygen (green) and silicon (red). (**a–c**) *Selaginella biformis* A. Braun ex Kuhn. (**d**) *S. ciliaris* (Retzius) Spring.

Table S1. Extant *Selaginella* species from Yunnan Province, Southwest China (modified from Chu, 2006).

No.	Taxon	Leaf type	Altitude (m)
1	*S. albocincta* Ching ex H.S. Kung	dimorphic	1900–2850
2	**S. amblyphylla* Alston	dimorphic	130–2300
3	**S. biformis* A. Braun ex Kuhn	dimorphic	130–1100
4	[&]*S. bisulcata* Spring	dimorphic	550–1950

5a	*S. bodinieri* var. *bodinieri* Hieron. ex Christ	dimorphic	1500–2050
5b	*S. bodinieri* var. *omeiensis* (Ching ex H.S. Kung) W.M. Chu	dimorphic	600–1300
6	*S. braunii* Bak.	dimorphic	400–1800
7	*S. chaetoloma* Alston	dimorphic	1150–2700
8	[&]*S. chrysocaules* (Hook. et Grev.) Spring	dimorphic	1500–2700
9	**S. ciliaris* (Retzius) Spring	dimorphic	130–1050
10	*S. compta* Hand. -Mazz.	dimorphic	650–2950
11	**S. decipiens* Warb.	dimorphic	1350–1500
12	**S. delicatula* (Desvaux ex Poiret) Alston	dimorphic	100–1500
13	**S. doederleinii* Hieronymus	dimorphic	300–1400
14a	*S. effusa* var. *dulongjiangensis* W.M. Chu	dimorphic	1440–1500
14b	**S. effusa* var. *effusa* Alston	dimorphic	120
15	**S. frondosa* Warb.	dimorphic	100–850
16	*S. gebaueriana* Hand. -Mazz.	dimorphic	800–2400
17	**S. helferi* Warburg	dimorphic	100–1000
18	*S. hengduanshanicola* W.M. Chu	dimorphic	2900–3200
19	*S. hezhangensis* P.S. Wang et X.Y. Wang	dimorphic	2400
20	[&]*S. heterostachys* Bak.	dimorphic	200–1900
21	*S. indica* (Milde) R.M. Tryon	monomorphic	1500–2500
22	[&]*S. involvens* (Swartz) Spring	dimorphic	500–2600
23	*S. jugorum* Hand. -Mazz.	dimorphic	2850–4025
24	[&]*S. kouycheensis* Lévl.	dimorphic	700–1300
25	[&]*S. kurzii* Bak.	dimorphic	650–2300
26	*S. labordei* Hieronymus ex Christ	dimorphic	800–2850
27	*S. laxistrobila* Shing	dimorphic	2600–3200
28	[&]*S. leptophylla* Bak.	dimorphic	950–2300
29	[&]*S. mairei* Lévl.	dimorphic	400–2200
30	[&]*S. megaphylla* Bak.	dimorphic	1300–1500

31	**S. minutifolia* Spring	dimorphic	500–680
32	&*S. moellendorffii* Hieronymus	dimorphic	350–1500
33a	**S. monospora* var. *monospora* Spring	dimorphic	1400–2400
33b	**S. monospora* var. *ciliolate* W.M. Chu	dimorphic	1600–2450
34	&*S. nipponica* Franchet et Savatier	dimorphic	380–880
35	**S. ornata* (Hooker et Greville) Spring	dimorphic	400–750
36	**S. petelotii* Alston	dimorphic	500–850
37	**S. pennata* (D. Don) Spring	dimorphic	400–1200
38a	**S. picta* f. *picta* A. Braun ex Baker	dimorphic	600
38b	&*S. picta* f. *viridis* Alston	dimorphic	100–1350
39	*S. prostrata* H.S. Kung	dimorphic	1750
40	*S. pulvinata* (Hooker & Greville) Maximowicz	dimorphic	1100–3000
41	**S. pseudopaleifera* Hand. -Mazz.	dimorphic	100–220
42	&*S. remotifolia* Spring	dimorphic	650–2600
43	**S. repanda* (Desv.) Spring	dimorphic	200–950
44	*S. rubella* W.M. Chu	dimorphic	2700–3000
45	*S. sanguinolenta* (L.) Spring	dimorphic	1750–3200
46	**S. siamensis* Hieronymus	dimorphic	800–1400
47	**S. superba* Alston	dimorphic	130–400
48	*S. trichophylla* Shing	dimorphic	1400–1900
49	&#*S. uncinata* (Desv.) Spring	dimorphic	1000–1100
50	*S. vardei* Lévl.	monomorphic	700–2700, ~3500
51	**S. willdenowii* (Desv.) Bak.	dimorphic	230–1500
52	*S. xichouensis* W.M. Chu	dimorphic	1500
53	&*S. xipholepis* Bak.	dimorphic	950–1300

*, (sub)tropical species; &, wide distribution with (sub)tropical occurrence; #, collected by the authors from Mengla County, Xishuangbanna Prefecture.

REFERENCES

Banks, J.A., et al., 2011. The *Selaginella* genome identifies genetic changes associated with the evolution of vascular plants. Science 332, 960–963. https://doi.org/10.1126/science.1203810.

Banks, J.A., 2009. *Selaginella* and 400 million years of separation. Annual Review of Plant Biology 60, 223–238. https://doi.org/10.1146/annurev.arplant.59.032607.092851.

Bek, J., Pšenička, J., Drábková, J., Zhou, W.M., Wang, J., 2023. *Thomasites* gen. nov. a new herbaceous lycophyte and its spores from late Duckmantian of the Radnice Basin, Czech Republic and palynological grouping of Palaeozoic herbaceous lycophytes. Review of Palaeobotany and Palynology 310, 104842. https://doi.org/10.1016/j.revpalbo.2023.104842.

Bienfait, A., Waterkeyn, L., 1974. Contribution à l'étude systématique des *Selaginella*. Spécificité des formations callosiques foliaires observées en fluorescence. Bulletin du Jardin botanique national de Belgique 44, 295–302.

Chu, W.M., 2006, Selaginellaceae. *in* Wu, C.Y. (ed.), Flora Yunnanica. Vol. 20. Beijing, Science Press, p. 35–93 (In Chinese).

Cremers, G., Boudrie, M., 2007. Two new species of *Selaginella* subgenus *Heterostachys* (Sellaginellaceae from The Guianas). Fern Gazette 18, 41–52.

Dahlen, M.A., 1988. Taxonomy of *Selaginella*: a study of characters, techniques, and classification in the Hong Kong species. Botanical Journal of the Linnean Society 98, 277–302. https://doi.org/10.1111/j.1095-8339.1988.tb01704.x.

Dengler, N.G., 1980. The histological basis of leaf dimorphism in *Selaginella martensii*. Canadian Journal of Botany 58, 1225–1234. https://doi.org/10.1139/b80-152.

Feng, Z., Lv, Y., Guo, Y., Wei, H.B., Kerp, H., 2017. Leaf anatomy of a late Palaeozoic cycad. Biology Letters 13, 20170456. https://doi.org/10.1098/rsbl.2017.0456.

Feng, Z., Wei, H.B., Guo, Y., He, X.Y., Sui, Q., Zhou, Y., Liu, H.Y., Gou, X.D., Lv, Y., 2020. From rainforest to herbland: New insights into land plant responses to the end-Permian mass extinction. Earth-Science Reviews 204, 103153.

https://doi.org/10.1016/j.earscirev.2020.103153.

Feng, Z., Sui, Q., Yang, J.Y., Guo, Y., McLoughlin, S., 2023. Specialized herbivory in fossil leaves reveals convergent origins of nyctinasty. Current Biology 33, 720–726. https://doi.org/10.1016/j.cub.2022.12.043.

Góes-Neto, L.A.A., Assis, E.L.M., Salino, A., 2017. *Selaginella* (Selaginellaceae) from Brazil: a new species, new records and lectotype designation. Kew Bulletin 72, 40. https://doi.org/10.1007/s12225-017-9714-5.

Góes-Neto, L.A.A., Heringer, G., Salino, A., 2015. *Selaginella salinoi* (Selaginellaceae), a new species from Brazil. Phytotaxa 224, 291–295. https://doi.org/10.11646/phytotaxa.224.3.8.

Góes-Neto, L.A.A., Salino, A., 2018. *Selaginella kriegeriana* (Selaginellaceae-Lycopodiopsida), an endemic new species from Brazil with notes about the genus in Caparáo National Park. Systematic Botany 43, 920–929. https://doi.org/10.1600/036364418X697643.

Guo, M.-q., Guo, J., Zhang, D.-w., Zhang, X.-c., Liu, B.-d., 2012. The systematic significance of foliar epidermis characteristic of nine species of *Selaginella* under SEM. Journal of Chinese Electron Microscopy Society 31, 163–168. https://doi.org/10.1007/s11783-011-0280-z.

Hsü, J., 1937. Anatomy, development and life history of *Selaginella sinensis* I. Anatomy and development of the shoot. Bulletin of Chinese Botanical Society 3, 75–95.

Liu, G., 1990. Permo-Carboniferous paleogeography and coal accumulation and their tectonic control in the North and South China continental plates. International Journal of Coal Geology 16, 73–117. https://doi.org/10.1016/0166-5162(90)90014-P.

Liu, L., Jie, D., Liu, H., Gao, G., Gao, Z., Li, D., Nannan, L., Guo, J., Qiao, Z., 2016. Assessing the importance of environmental factors to phytoliths of *Phragmites communis* in North-Eastern China. Ecological Indicators 69, 500–507. https://doi.org/10.1016/j.ecolind.2016.05.009.

Lopes, L.K.C., Feio, A.C., 2020. Silica bodies in *Selaginella* (Selaginellaceae). American

Fern Journal 110, 29–41. https://doi.org/10.1640/0002-8444-110.1.29

Matsunaga, K.K.S., Cullen, N.P., Tomescu, A.M.F., 2017. Vascularization of the *Selaginella* rhizophore: anatomical fingerprints of polar auxin transport with implications for the deep fossil record. New Phytologist 216, 419–428. https://doi.org/doi:10.1111/nph.14478.

Scotese, C.R., 2021. An atlas of Phanerozoic paleogeographic maps: The seas come in and the seas go out. Annual Review of Earth and Planetary Sciences 49, 679–728. https://doi.org/10.1146/annurev-earth-081320-064052.

Shen, S.Z., et al., 2019. Permian integrative stratigraphy and timescale of China. Science China Earth Sciences 62, 154–188.

Sun, Z.Y., Zhang, X.C., Cui, S.M., Zhou, F.Q., 2006. Leaf morphology of 29 Chinese and one Thailand species of the Selaginellaceae and its taxonomic significance.Acta Phytotaxonomica Sinica 44, 148–160. https://doi.org/10.1360/aps040110.

Šimůnek, Z., Thomas, B.A., 2012. A new species of *Selaginella* (Selaginellaceae) from the Bolsovian (Carboniferous Period) of the Zonguldak – Amasra Coal Basin, north-western Turkey. Geologia Croatica 65, 345–350.

Thomas, B.A., 2005. A reinvestigation of *Selaginella* species from the Asturian (Westphalian D) of the Zwickau coalfield, Germany and their assignment to the new sub-genus *Hexaphyllum*. Zeitschrift Der Deutschen Gesellschaft Für Geowissenschaften 156, 403–414. https://doi.org/10.1127/1860-1804/2005/0156-0403.

Valdespino, I.A., 2015a. *Selaginella boomii* (Selaginellaceae–Lycopodiophyta): A new and widely distributed spikemoss from South America. Brittonia 67, 328–335.

Valdespino, I.A., 2015b. Lectotypification of *Selaginella tenuissima* and *S. papagaiensis* (Selaginellaceae) with the description of *S. monticola*. Phytotaxa 233, 153–165. https://doi.org/10.11646/phytotaxa.233.2.3.

Valdespino, I.A., 2015c. Novelties in *Selaginella* (Selaginellaceae–Lycopodiophyta), with emphasis on Brazilian species. PhytoKeys 57, 93–133. https://doi.org/10.3897/phytokeys.57.6489.

Valdespino, I.A., 2017a. *Selaginella hyalogramma* (Selaginellaceae–Lycopodiophyta):

a new species from Venezuela, South America. American Fern Journal 107, 72–83. https://doi.org/10.1640/0002-8444-107.2.72

Valdespino, I.A., 2017b. Validation of *Selaginella psittacorrhyncha* (Selaginellaceae), a new species from the Guiana Highlands of Venezuela and Brazil. Phytoneuron 63, 1–8.

Valdespino, I.A., 2017c. Novel fern- and centipede-like *Selaginella* (Selaginellaceae) species and a new combination from South America. PhytoKeys 91, 13–38. https://doi.org/10.3897/phytokeys.91.21417.

Valdespino, I.A., Heringer, G., Salino, A., Góes-Neto, L.A.A., Ceballos, J., 2015. Seven new species of Selaginella subg. *Stachygynandrum* (Selaginellaceae) from Brazil and new synonyms for the genus. PhytoKeys 50, 61–99. https://doi.org/10.3897/phytokeys.50.4873.

Valdespino, I.A., López, C.A., Ceballos, J., 2018a. *Selaginella germinans* (Selaginellaceae), a new articulate species from Chapada dos Veadeiros region in the State of Goiás. Botany Letters 165, 487–493. https://doi.org/10.1080/23818107.2018.1496849.

Valdespino, I.A., López, C.A., Ceballos, J., 2018b. From the Guiana Highlands to the Brazilian Atlantic Rain Forest: four new species of *Selaginella* (Selaginellaceae–Lycopodiophyta: *S. agioneuma*, *S. magnafornensis*, *S. ventricosa*, and *S. zartmanii*). PeerJ 6, e4708. https://doi.org/10.7717/peerj.4708.

Valdespino, I.A., López, C.A., Góes-Neto, L.A.A., 2014. Additions to Cuban *Selaginella* (Selaginellaceae). Phytotaxa 184, 235–244.

Wang, J., Shao, L.Y., Wang, H., Spiro, B., Large, D., 2019. SHRIMP zircon U–Pb ages from coal beds across the Permian–Triassic boundary, eastern Yunnan, southwestern China. Journal of Palaeogeography7, p. 117–129. https://doi.org/10.1016/j.jop.2018.01.002.

Wang, J.D., Li, H.M., 1998. Paleo-latitude variation of Guizhou terrain from Devonian to Cretaceous. Chinese Journal of Geochemistry 17, 356–361.

Weststrand, S., Korall, P., 2016. Phylogeny of Selaginellaceae: There is value in morphology after all! American Journal of Botany 103, 2136–2159.

https://doi.org/10.3732/ajb.1600156.

Yang, P., Zhang, X.C., 2003. Studies on the Chinese Selaginellaceae V: Observations on the foliar epidermis of *Selaginella* Beauv. from Hainan Island in China. *in* Chandra, S., Srivastava, M. (eds.), Pteridology in the New Millennium. Dordrecht, Kluwer Academic Publishers, p. 177–184.

Zhang, X.C., Nooteboom, H.P., Kato, M., 2013. Selaginellaceae. *in* Wu, Z.Y., Raven, P.H., Hong, D.Y. (eds.), Flora of China, Vol. 2–3 (Pteridophytes). Beijing, Science Press; St. Louis, Missouri Botanical Garden Press, p. 37–66.

Zhao, X.H., Mo. Z.G., Zhang, S.Z., Yao, Z.Q., 1980. Late Permian flora in western Guizhou and eastern Yunnan. *in* Nanjing Institute of Geology and Palaeontology, Academia Sinica (ed.), Stratigraphy and Palaeontology of upper Permian coal-bearing formation in western Guizhou and eastern Yunnan. Beijing, Science Press, p. 70–122 (In Chinese).

Zhou, X.M., et al., 2016. A large-scale phylogeny of the lycophyte genus *Selaginella* (Selaginellaceae: Lycopodiopsida) based on plastid and nuclear loci. Cladistics 32, 360–389. https://doi.org/10.1111/cla.12136.
